# The Patient’s Journey in Obesity within the United States: An Exercise of Resilience against Disease

**DOI:** 10.3390/life14091073

**Published:** 2024-08-27

**Authors:** Kayla Northam, Malikiya Hinds, Sreevidya Bodepudi, Fatima Cody Stanford

**Affiliations:** 1 MGH Weight Center, Massachusetts General Hospital, Boston, MA 02114, USA; knortham@mgb.org (K.N.); sbodepudi@mgh.harvard.edu (S.B.); 2 Department of Medicine, Dartmouth College, Lebanon, NH 03756, USA; 3 Department of Gastroenterology, Dartmouth Hitchcock Medical Center, Lebanon, NH 03756, USA; 4 Harvard Medical School, Boston, MA 02114, USA; malikiya.a.hinds@gmail.com; 5 Nutrition Obesity Research Center, Harvard Medical School, Boston, MA 02114, USA; 6 Department of Medicine-Neuroendocrine Unit and Department of Pediatrics-Endocrinology, Massachusetts General Hospital, Harvard Medical School, Boston, MA 02114, USA

**Keywords:** obesity, pharmacotherapy, bariatric surgery, barriers to care

## Abstract

Obesity is often viewed as a result of patient failure to adhere to healthy dietary intake and physical activity; however, this belief undermines the complexity of obesity as a disease. Rates of obesity have doubled for adults and quadrupled for adolescents since the 1990s. Without effective interventions to help combat this disease, patients with obesity are at increased risk for developing type 2 diabetes, heart attack, stroke, liver disease, obstructive sleep apnea, and more. Patients often go through several barriers before they are offered pharmacotherapy or bariatric surgery, even though evidence supports the use of these interventions earlier. This partially stems from the cultural barriers associated with using these therapies, but it is also related to healthcare provider bias and limited knowledge of these therapies. Finally, even when patients are offered treatment for obesity, they often run into insurance barriers that keep them from treatment. There needs to be a cultural shift to accept obesity as a disease and improve access to effective treatments sooner to help decrease the risk of health complications associated with obesity.

## 1. Introduction

Obesity is a chronic disease with rising rates across the globe. The World Health Organization (WHO) has found that 1 in 8 adults worldwide have obesity [1,2]. Rates of obesity have doubled for adults and quadrupled for adolescents since the 1990s. Without effective interventions to help combat this disease, patients with obesity are at increased risk for developing type 2 diabetes, heart attack, stroke, liver disease, obstructive sleep apnea, and more [3,4].

While the exact body mass index (BMI) qualifications for overweight and obesity can vary based on age and ethnicity (and these are controversial since BMI does not correlate to metabolic health) [2,5], patients are considered to be overweight with a body mass index (BMI) greater than or equal to 25 and considered to have obesity with a BMI greater than 30 [3]. Patients who have health complications such as hypertension, hyperlipidemia, diabetes, and obstructive sleep apnea are eligible for obesity treatments at a BMI of 27 or greater, despite the lower BMI. There are some issues with the accuracy of BMI, but it is still a helpful tool for identifying patients at greater risk for health complications due to excess body fat [2,3,5].

**Figure 1:**
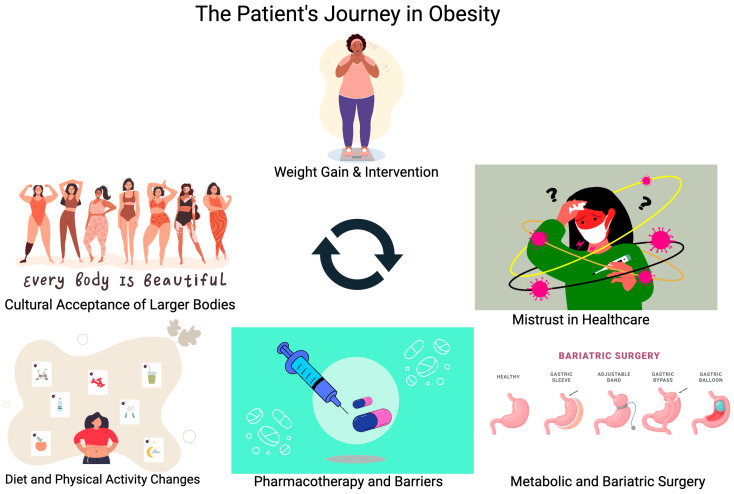
Illustrating the different factors contributing to a patient’s journey to treat obesity.

This paper will focus on the journey patients with obesity undergo while struggling to find effective therapy for their disease. By the time patients are seeking help from their primary care office or obesity medicine specialists, many have already tried and failed dietary and exercise management of this disease. Currently, only 1–2% of adults in the United States who qualify for pharmacotherapy or surgery for management of obesity are offered these treatment options, despite evidence that this is far more effective compared to lifestyle management alone [4,6]. Recognizing and appreciating the effort patients have put into managing this chronic illness is an important step in helping to decrease patient fear of weight bias from their healthcare providers. Treatment of obesity needs to include a variety of interventions beyond dietary and lifestyle modification, as outlined in Figure 1. These other interventions should be introduced earlier rather than withholding treatment until lifestyle modification is achieved.

## 2. Weight Gain and Early Intervention

Obesity is a complex chronic health illness that can develop due to a variety of factors [7]. Genetics, dietary intake, physical activity, environmental factors, race, age, gender, and medications are all known reasons why patients could develop obesity during their lifespan [8,9]. However, there is a cultural stigma against patients with obesity, where dietary intake and physical activity are seen as the primary drivers of obesity. Evidence shows the opposite: environmental and genetic factors appear to be the primary risk factors for obesity [10].

During the phase of initial weight gain in patients who have obesity, the treatment typically focuses on the behavioral and environmental factors that contribute to obesity. Dietary changes and increased physical activity are meant to help manage these aspects of the disease [8]; however, obesity and the risk of developing obesity must be recognized and diagnosed in the patient before they can receive medical assistance with this [9,11]. This is challenging in the primary care setting for many reasons, and the younger a patient is, the more difficult it is for patients to receive appropriate care [12].

In one article from 2019, 206 of 280 physicians completed a survey regarding BMI assessment and interventions used in the primary care setting. Only 61% discuss BMI with their patients routinely, even though 91% of them calculate it during the visit. Of the physicians who do discuss BMI with their patients, they are primarily addressing dietary changes and increased physical activity. They are far less likely to start pharmacotherapy or refer for surgical interventions [11,13]. This is likely due to limited knowledge and training within health care providers on the intricacies of obesity management [14].

Identification of obesity and management is even more challenging within the pediatric setting. In one literature review, weight stigma and bias were investigated for pediatric patients with obesity. Pediatric patients who were seeking help for obesity were more likely to experience stigma and bullying. This contributes to the cycle of worsening mood disorders and the risk of developing eating disorders in their attempts to manage obesity. Like with adult patients with obesity, much of this struggle stems from the belief that obesity is a personal failing rather than a complex disease with various etiologies [10,12].

The impact of weight stigma stretches beyond their peers among pediatric patients. Many patients experience weight stigma from their healthcare providers, which increases their risk of either avoiding future appointments (24%) or seeking a new healthcare provider (35%). This leads to further delay in effective treatment for these patients, which in turn impacts the ability to prevent health complications related to obesity [10,12].

## 3. A Cultural Shift toward Acceptance

While healthcare providers are using BMI and waist circumference to help determine patients who need assistance with improving their overall health risks, it is also important to cultivate a positive body image. A positive body image has been found to be a critical component in determining a person’s ability to reach weight loss goals [15], and contemporary culture has shown progression toward more body positivity, self-empowerment, inclusivity, and encouraging individuals’ acceptance and pride in their bodies, despite BMI’s classifying them as clinically overweight or obese [16].

This cultural phenomenon has been widely popularized through social media via platforms like Instagram, with more than 11 million posts tagged with #bodypositive, 4 million for #bodypositivity, and over 1 million for #bopo [17]. The body positivity movement (BPM) challenges predominant body image ideals within the United States, promoting acceptance and respect of all bodies, irrespective of shape, size, and features. BPM focuses more on the appreciation of the functionality and health of the body than solely on its appearance [17].

Critics argue that body positivity was initially aimed at helping groups such as cancer survivors dealing with physical disfigurements, individuals with physical disabilities, and racially/ethnically underrepresented people often overlooked by the media. These groups are distinguished by physical characteristics over which they have no control [16].

Does BPM lead to a higher prevalence of obesity? According to Muttarak (2018), increased visual normalization of larger bodies via increased habitual visual exposure to people with excess weight may have implications for further contributing to the higher prevalence of overweight and obesity, particularly among people with lower levels of education and income [18].

Like the BPM, the fat acceptance movement (FAM) was established as a supporting movement that protested workplace discrimination and the lack of fat acceptance in society [19]. The movement was dedicated to preserving rights and improving the quality of life for “fat people” [20].

The BPM and FAM have been fundamental in increasing media representation and helping reduce obesity stigmatization [16]. With the impetus of the movement, terms like “fat”, “curvy”, “plus-sized”, and “full-figured” have become more frequently used among plus-size fashion bloggers, prevailing the reclamation of the use of the word “fat” and contributing to the reduction of the weight stigma surrounding obesity [21].

The positive aspect of the BPM and FAM is that there have also been policy shifts towards body acceptance, which has helped craft more inclusive environments and positive perceptions of overweight and obese people by lawmakers [16]. Anti-discrimination law addresses injustice arising from governmental categorization of certain groups that are targeted and disadvantaged without adequate justification or in employment contexts where decisions are made based on the protected characteristics specified in Title VII of the 1964 Civil Rights Act. Advocates for the rights of overweight individuals understand the challenge of making legal cases for the protection of rights for those with excess weight under Title VII, especially while concurrently framing obesity as a medical impairment and disability [21,22,23]. These actions are crucial for improving the quality of life for patients with obesity.

Despite the positive changes from BPM and FAM, it becomes more challenging for healthcare providers to address overweight and obese patients. There is even a wave of patients bringing cards saying “don’t weigh me” to their health appointments [21]. Sentiments like “fit, fabulous, and fat” lifestyles served to contradict the public health messaging discouraging unhealthy lifestyles [16]. One must ask: does this contribute to a worsening prevalence of obesity? This is an important consideration among people with central adiposity, potentially undermining the recognition of being overweight and its health consequences among patients who struggle with this chronic disease [18].

## 4. Health at Every Size

Another movement is HAES^®^ (Health at Every Size). This promotes a weight-neutral perspective, supporting the notion that one can attain and maintain health regardless of weight. This approach encourages intuitive eating, a method that focuses on responding to one’s internal hunger signals and feelings of fullness, as well as personal food preferences [15]. In contrast, the BPM and FAM do not necessarily encourage healthy eating habits and physical activity.

HAES^®^ aims to tackle weight bias and stigma in patients with obesity, promoting itself as an effective public health strategy that shifts focus away from weight as the primary indicator of health [15]. The approach is also valued for its rejection of restrictive dieting, which has been linked to higher psychological stress, elevated cortisol levels, and eventual weight gain [24]. HAES^®^ supports the notion of fit at every size.

The HAES^®^ (Health at Every Size) approach not only advocates for acceptance but also encourages pride in the aesthetic of larger bodies [16]. Participants in this movement aim to reintegrate people of larger sizes into the mainstream of society, strongly denouncing those who disagree with their views as “body-shamers” and “fat-phobic”, accusing them of upholding conventional societal standards [16].

HAES^®^ could potentially decrease the likelihood that a patient with obesity will seek effective treatment [16]. Despite extensive empirical medical and comprehensive epidemiological research, numerous fat activists proudly embrace their higher BMI and larger size, often viewing efforts to promote health and wellness with skepticism and disdain. Years of medical and scientific research have unequivocally demonstrated the adverse consequences of not treating obesity in over 650 million overweight and obese individuals globally [16]. At the same time, it is crucial that the medical and public health communities do not leverage efforts to reduce obesity as a pretext for discrimination and oppression. Overweight and obese patients are entitled to their own choices; however, ensuring they have health literacy and access to accurate information to make informed health decisions is a critical public health priority [16].

The study by Ulian (2018) investigated the effects of an intensive Health at Every Size^®^ (HAES^®^) intervention on various aspects of health in women with obesity [25]. Conducted over seven months, the study employed a randomized controlled trial format, with one group undergoing the intensive HAES^®^ intervention (I-HAES^®^) and another undergoing a traditional HAES^®^ intervention (CTRL). Physiological, psychological, and behavioral parameters were assessed before and after the interventions using both quantitative and qualitative methods [25].

Results of the study indicated that while there were no significant changes in body weight or physical activity levels, the I-HAES^®^ group showed improvements in peak oxygen uptake, performance in a timed-stand test, eating attitudes, and body image perception [25]. Additionally, the I-HAES^®^ group experienced greater enhancements in health-related quality of life, particularly in physical and psychological health domains, compared to the CTRL group [25]. These findings suggest that the intensified HAES^®^ intervention yielded superior outcomes in various health aspects, despite the absence of significant changes in weight or physical activity levels [25]. To determine if this is enough to mitigate the long-term risks of adverse health events associated with obesity, these interventions and groups would need to be assessed across their lifespan. Unfortunately, it would be a challenging undertaking to accomplish this.

## 5. Mistrust in Healthcare

Another barrier patients must overcome to seek effective therapy for obesity is the innate mistrust in healthcare within the United States, especially among vulnerable populations [26]. This mistrust stems from historical violations of trust and unethical medical research, such as the infamous Tuskegee Study, as well as a sense that patients might not be receiving effective care [26,27]. Much of this mistrust, especially for marginalized groups, is warranted and appropriate, but presents a challenge for healthcare providers to address before patients might be willing to address health concerns or seek therapy [26].

Social media can often promote medical mistrust and misinformation [28], and it can be challenging for patients to determine if the information they are being told is true or false [29]. Compounding this issue are the attacks against physicians and other healthcare providers who try to combat the misinformation being spread [28]. A clear example is the misinformation and mistrust regarding the COVID-19 pandemic and vaccine [30]. This has led to hesitancy for marginalized groups, such as black Americans, to have access to adequate therapies for treating the virus [31].

## 6. Dietary Changes and Physical Activity

Traditionally, the first line of therapy for obese or overweight patients is lifestyle modification, including dietary changes and increasing physical activity [32]. When patients present to their primary care provider’s office, dietary changes are more likely to be suggested compared to other interventions (such as pharmacologic or surgical). This could be related to the perception of insufficient knowledge for utilizing pharmacotherapy or surgical intervention [33,34], but dietary changes and physical activity alone are not sufficient for the management of obesity.

Many commercial weight loss programs are available for patients that promise a quick fix for weight loss, but the results are often not sustainable. The other problem is that the concept of creating a calorie deficit disregards the complex nature of obesity and overweight as a chronic health condition [32]. This is why dietary modification alone will not achieve sufficient, sustainable weight loss for patients. Since this is the standard recommendation for helping patients manage their obesity, lifestyle modification becomes an obstacle for patients to overcome before they can reach effective therapies.

This is not to say that modifying diet and increasing physical activity is not an important piece of managing obesity; however, it needs to be adjunctive therapy rather than solo management. There have been research findings that support how low-calorie dietary changes can have a positive influence on the inflammatory process, which can contribute to the development and worsening of obesity.

Bosch-Sierra and colleagues [35] looked at the impact of a low-calorie diet on inflammatory markers and insulin resistance for both those with obesity and those with and without good metabolic health. Seventy-four subjects underwent 2–6-week cycles of the very low-calorie diet (VLCD) for a total of 12 weeks.

Subjects were selected from those seeking treatment for obesity within the Department of Endocrinology of the University Hospital in Valencia, Spain. They must have maintained a stable weight with a BMI > 30 for the past 5+ years. Subjects with obesity and poor metabolic health were identified if they displayed evidence of metabolic syndrome: dyslipidemia, elevated systolic or diastolic BP (blood pressure), impaired fasting glucose, or elevated A1C. Increased waist circumference was considered for both those with obesity and those with and without good metabolic health [35].

The VLCD consisted of liquid nutrition to supplement their dietary intake. This was performed through the use of Optisource Plus by Nestle. This gave the participants 654 kcal per day to replace their three primary meals. Serology was assessed at baseline and 6 months post intervention. This included the following: lipid profile, nutritional status, liver/renal function, blood count, coagulation, and hormonal profile. Additionally, the following inflammatory markers were assessed: proinflammatory cytokines and adipokines, oxidative stress by flow cytometry assay, and total antioxidant capacity [35].

Ultimately, the subjects experienced a 14.3 kg (+/−15.6 kg) weight reduction and a 16% fat mass index reduction. Visceral fat decreased by 32%. The lipid profile, A1C, and inflammatory markers all showed improvement. Both groups showed improvement in their adipokine levels without an increase in their interleukin profile. The changes were more pronounced in subjects with obesity and poor metabolic health compared to subjects with obesity and good metabolic health [35].

The problem with relying on VLCD as a solo therapy for obesity management is the sustainability of weight loss and the risk of weight cycling. The metabolic adaptation associated with VLCD also makes repeat attempts at weight loss more challenging for patients. This is why dietary modification for the management of obesity is rarely a long-term solution for patients [36]. The body resists weight loss attempts by reducing energy expenditure.

## 7. Pharmacotherapy

Over the past 12 years, the efficacy of anti-obesity medications has increased dramatically but is under-prescribed across the United States, even though 50% of adults qualify for therapy [9]. Qsymia (phentermine/topiramate) received FDA approval in 2012, and Contrave (bupropion/naltrexone) in 2014. These medications have several limitations based on patients’ comorbid conditions, such as cardiovascular disease (they would need to avoid stimulants such as phentermine within Qsymia (phentermine/topiramate)) and the need for opioid therapy (they would need to avoid naltrexone within Contrave) [37]. Additionally, the side effects lead to a high dropout rate (patients stopping medication). According to the findings of Gadde et al. (2020), the dropout rate for Qsymia is 17.5% and Contrave is 24%. Weight loss with these is also significantly lower than desired with these medications, with an average of 5.4% for Contrave or 10.7% for Qsymia [37].

GLP-1 RA (glucagon-like peptide-1 receptor agonists) medications such as liraglutide (Saxenda) or semaglutide (Wegovy), or dual agonist GLP-1/GIP (glucose-dependent insulinotropic polypeptide) RA, tirzeptide (Zepbound), have made substantial weight loss achievable. For patients who are not able to or are reluctant to obtain metabolic and bariatric surgery (MBS), the use of these medications offers a chance for success that they might not have been able to achieve [38]. These medications have shown benefits for improving cardiovascular risk factors [39], decreasing obesity-associated liver disease [40], and kidney disease [41]. Semaglutide has proven so effective at reducing cardiovascular risk that it was recently approved by the FDA for this indication [42], and it will not be surprising if additional indications are approved by the FDA in the future. Additionally, the weight loss associated with using these medications can help decrease pressure and joint injuries. Research has shown that a weight loss of 5.1 kg reduces the risk of osteoarthritis by 50% [43].

The expected weight loss from injectable medications is much higher compared to their oral counterparts. They also do not have as many contraindications. Other than intolerance or side effects to the medications, the only contraindications are pregnancy, personal history of pancreatitis, or family history of multiple endocrine neoplasia syndrome type 2 (MEN2) or medullary thyroid cancer (MTC) [44]. This makes injectable medications accessible to a wider range of patients, so long as they are financially feasible.

The available GLP-1 RAs for weight loss are liraglutide (Saxenda) and semaglutide (Wegovy). Liraglutide (Saxenda) produces an average of 6.5% weight loss [45]. This weight loss is slightly less than phentermine/topiramate (Qsymia); however, it does not have the same degree of contraindications, and it has shown cardiovascular benefit, like semaglutide (Wegovy) [45]. Patients discontinuing use of liraglutide (Saxenda) due to side effects are comparable to phentermine/topiramate (Qsymia) and buproion/naltrexone (Contrave) at 14% [46]. For semaglutide, the average expected weight loss is 14.5% [35], and discontinuation due to side effects is 16.6% [39].

For tirzepatide (Zepbound), which is the first available combination GLP-1/GIP receptor agonist, expected weight loss increases to 20%. Even more appealing to patients beyond weight loss is the lower rate of discontinuation of medication due to side effects. With tirzepatide (Zepbound), this is 3–6% [47].

## 8. Barriers to Pharmacotherapy

Unfortunately for patients, there are limitations on being able to start or continue the use of pharmacotherapy for obesity or overweight. First is finding a healthcare provider who is comfortable prescribing medications to treat obesity [6,9,14,48]. In a study by Oshman et al. (2023), less than one third of physicians prescribed anti-obesity medications. The majority refer patients to local resources such as WW (Weight Watchers).

The next barrier patients face within the United States is insurance coverage [9,49] for all forms of pharmacotherapy, but especially for injectable therapies. With the out-of-pocket cost for liraglutide (Saxenda), semaglutide (Wegovy), and tirzepatide (Zepbound) ranging from USD 900–1400 per month, insurance coverage for many patients is critical for being able to obtain treatment; however, coverage for these medications varies greatly depending on the patient’s individual insurance plan [50]. Medicare and Medicaid still do not cover the use of obesity pharmacotherapy. The only exception to this has been the recent coverage of Wegovy for cardiovascular risk reduction, but obesity alone is not covered [4]. Expansion of Medicare and Medicaid coverage has shown to decrease health disparities across the U.S., so additional expansion to include management of obesity could improve health outcomes for individuals with this disease [51].

Even when patients do have insurance coverage, they often must obtain a prior authorization, which is time-consuming for the healthcare provider and must be completed correctly to be approved. In some cases, the need for prior authorization will influence a provider’s therapy choices for patient care [52]. Then, if patients are able to obtain the medication through insurance, success is currently measured by the percentage of weight loss. So patients could lose coverage if they do not achieve this, which does not take into consideration other factors that could be limiting the degree of weight loss. If patients require the addition of medications that promote weight gain, this could mask the impact of these medications and lead to a loss of coverage [53].

For some of the less expensive medications available to treat obesity, a patient’s past medical history or other medications might limit what can be used [37]. Each oral medication, such as phentermine/topiramate (Qsymia), bupropion/naltrexone (Contrave), or their individual counterparts (phentermine, topiramate, bupropion, and naltrexone), has contraindications where the medication must be avoided. These contraindications are more common compared to those of GLP-1 RA therapies [37].

With the cost of injectable therapies when they are not covered by insurance and the limitations associated with oral obesity medications, it is no surprise that patients have turned towards the use of compounding pharmacies. This stems from their desperation to obtain treatment [54]. Patients using compounding pharmacies are at increased risk for serious health complications since there is no guarantee they are obtaining the correct medication at the correct dose and that it is not contaminated [54]. The Obesity Medicine Association has released a statement against the use of compounding pharmacies since the only authorized retailers of anti-obesity injections are Eli Lilly or Novo Nordisk [42]. Patients using compounding pharmacies are at increased risk for serious health complications since there is no guarantee they are obtaining the correct medication at the correct dose and that it is not contaminated [54].

## 9. Metabolic and Bariatric Surgery (MBS)

For many patients, obtaining coverage for metabolic and bariatric surgery (MBS) can be easier compared to available pharmacotherapy. Based upon the updated guidelines from ASMBS (2022) [55], when patients have a BMI > 30 with a health complication such as type 2 diabetes, non-alcoholic steatohepatitis, non-alcoholic fatty liver disease, obstructive sleep apnea, hypertension, or a BMI > 35 regardless of health complications, bariatric surgery should be covered by insurance. While insurance policies have not commonly accepted these new recommendations, we would expect to see a shift towards this in the future [55].

Unfortunately, from a patient’s perspective, surgery is often seen as a last resort option for patients when they fail to achieve weight loss through other methods rather than an effective therapy for chronic disease [56,57]. This is especially true for adolescent patients, where it is viewed as too permanent or invasive, despite evidence clearly showing it is a safe and effective tool for managing obesity [58,59]. Less than 0.1% of eligible adolescents receive MBS for the management of obesity [59,60]. Compared to adults who receive MBS, adolescents typically have a larger weight reduction and a greater improvement in health complications [61].

Another trend that patients with obesity must overcome is weight regain after bariatric surgery [62]. Weight regain [62,63] occurs with all subtypes of bariatric surgery, so medication therapy is necessary for continued management of obesity [64,65,66]. “Weight loss pharmacotherapy serves as a useful adjunct to bariatric surgery in patients with inadequate weight loss or weight regain” [64]. Since sleeve gastrectomy is the most common bariatric surgery per the numbers released by the American Society for Metabolic and Bariatric Surgery (ASMBS) [67], addressing weight recurrence or weight regain after surgery is something that healthcare providers will need to be comfortable managing.

The weight recurrence patients can experience after bariatric surgery only returns them to the cycle of frustration they dealt with prior to surgery, especially if they cannot realistically obtain the most effective pharmacotherapies available due to supply shortages or insurance barriers. Research for adolescents with weight regain after MBS has shown that oral medication options—such as phentermine, topiramate, and metformin—are effective to help combat this [68]. One study looked at the effectiveness of dietary measures or GLP-1 RA therapy to treat weight recurrence after bariatric surgery. Not surprisingly, the GLP-1 RA therapy was far superior in helping patients manage weight recurrence [65,69,70]. This was true regardless of the type of surgery.

## 10. Conclusions

Recently, there has been a sudden boom in the management of obesity with the approval of medications such as liraglutide, semaglutide, and tirzepatide. Additionally, metabolic and bariatric surgery remain available to help patients effectively combat the disease of obesity [13]. Still, the road for patients to find providers who will manage obesity effectively is long and full of obstacles to overcome. The primary method of obesity management utilized across the U.S. is dietary modification and increased physical activity, but evidence does not support using this lifestyle modification as solo therapy for this disease [71]. The limited or poor results of using lifestyle modification can be highly discouraging for patients struggling with this disease [72].

These barriers from society, providers, and insurance policies within the United States contribute to the delay in utilizing effective therapy. Less than 1% of eligible patients receive treatment for obesity [6]. Social media and cultural trends such as the body positivity movement and fat acceptance movements might contribute to patients avoiding treatment, such as refusing to be weighed during health visits in an attempt to avoid biased treatment. Healthcare providers might become less comfortable with addressing excess body weight and the health implications associated with it. Or the healthcare providers who do address obesity and excess body weight might not be confident in utilizing pharmacotherapy or surgery [14,33,34].

Delays in effective treatment can shorten patient lifespans and lead to additional obesity-related complications. Even now, with the recent approval for the use of semaglutide for cardiovascular risk reduction, it is extremely challenging for patients to obtain coverage in a timely fashion [42].

Additionally, the barriers explored within this paper do not include the reality of medication shortages that have been ongoing for the past several years. So even when patients are able to try dietary modification, then find a provider to recognize their obesity, and then have that provider prescribe effective treatment and obtain insurance coverage, they may not be able to start therapy [73].

The most effective management for obesity is a multidisciplinary approach and early intervention. This includes dietary changes (without necessarily starting a low-calorie diet), increased physical activity, and the addition of pharmacotherapy or surgery as appropriate. Increasing the awareness and comfort level of primary care offices about using these medications will decrease the delay in patients receiving reliable access to care. A cultural shift accepting obesity as a disease and an increase in public outcry for coverage of medications will also be necessary to keep improving the management of obesity [74]. Patients with obesity have had to overcome overwhelming odds in order to treat their disease. We need to help improve health equity by decreasing these healthcare barriers.

## References

[1] Obesity and Overweight. https://www.who.int/news-room/fact-sheets/detail/obesity-and-overweight#:~:text=Obesity%20is%20a%20chronic%20complex,the%20risk%20of%20certain%20cancers.

[2] Narayan A., Agarwal A.A., Stanford C.F. (2024). Challenges with Relying on Body Fat and Weight Values for Obesity—Reply. JAMA Intern. Med..

[3] Overweight and Obesity. https://www.cdc.gov/obesity/php/about/index.html.

[4] Waldrop W.S., Johnson R.V., Stanford C.F. (2024). Inequalities in the provision of GLP-1 receptor agonists for the treatment of obesity. Nat. Med..

[5] Kyle K.T., Nadglowski F.J., Stanford C.F. (2020). Weight Management and Healthy Lifestyles. JAMA Intern. Med..

[6] Claridy D.M., Czepiel S.K., Bajaj S.S., Stanford C.F. (2021). Treatment of Obesity: Pharmacotherapy Trends of Office-Based Visits in the United States from 2011 to 2016. Mayo Clin. Proc..

[7] What Are Overweight and Obesity?. https://www.nichd.nih.gov/health/topics/obesity/conditioninfo/cause.

[8] Ghosh S., Bouchard C. (2017). Convergence between biological, behavioural and genetic determinants of obesity. Nat. Rev. Genet..

[9] Laddu D., Neeland J.I., Carnethon M., Stanford C.F., Mongraw-Chaffin M., Gibbs B.B., Ndumele E.C., Longenecker T.C., Chung L.M., Rao G. (2024). Implementation of Obesity Science into Clinical Practice: A Scientific Statement from the American Heart Association. Circulation.

[10] Washington B.T., Johnson R.V., Kendrick K., Ibrahim A.A., Tu L., Sun K., Stanford C.F. (2023). Disparities in Access and Quality of Obesity Care. Gastroenterol. Clin. N. Am..

[11] Hite A., Victorson D., Elue R., Plunkett B.A. (2019). An Exploration of Barriers Facing Physicians in Diagnosing and Treating Obesity. Am. J. Health Promot..

[12] Haqq A.M., Kebbe M., Tan Q., Manco M., Salas X.R. (2021). Complexity and Stigma of Pediatric Obesity. Child. Obes..

[13] Stanford C.F. (2024). A new era in obesity management. Nat. Rev. Gastroenterol. Hepatol..

[14] Olson A., Stanford C.F., Butsch S.W. (2023). Obesity in the USMLE Step 1 examination: A call to action. Int. J. Obes..

[15] Penney T.L., Kirk S.F. (2015). The Health at Every Size paradigm and obesity: Missing empirical evidence may help push the reframing obesity debate forward. Am. J. Public Health.

[16] McWhorter K.L. (2020). Obesity Acceptance: Body Positivity and Clinical Risk Factors. Cardiac Diseases—Novel Aspects of Cardiac Risk, Cardiorenal Pathology and Cardiac Interventions.

[17] Cohen R., Newton-John T., Slater A. (2021). The case for body positivity on social media: Perspectives on current advances and future directions. J. Health Psychol..

[18] Muttarak R. (2018). Normalization of Plus Size and the Danger of Unseen Overweight and Obesity in England. Obesity.

[19] Rasmussen W.D. (2019). The Rights/Development Nexus: Sen, Olson, and the Obesity Rights Movement. Soc. Sci. Q..

[20] NAAFA About Us. https://naafa.org/aboutus.

[21] Kirkland A. (2008). Think of the Hippopotamus: Rights Consciousness in the Fat Acceptance Movement. Law Soc. Rev..

[22] Reyes C.J.K., Sabharwal S., Stanford C.F. (2023). Legal Evolution of a Law against Weight Discrimination in the United States: A Focus on Massachusetts. Am. J. Health Promot..

[23] Sabharwal S., Reyes C.J.K., Stanford C.F. (2020). Need for Legal Protection against Weight Discrimination in the United States. Obesity.

[24] Lucassen A.E., Cizza G. (2012). The Hypothalamic-Pituitary-Adrenal Axis, Obesity, and Chronic Stress Exposure: Sleep and the HPA Axis in Obesity. Curr. Obes. Rep..

[25] Dimitrov Ulian M., Pinto A.J., de Morais Sato P., Benatti F.B., Lopes de Campos-Ferraz P., Coelho D., Roble O.J., Sabatini F., Perez I., Aburad L. (2018). Effects of new intervention based on Health at Every Size approach for the management of obesity: The “Health and Wellness in Obesity” study. PLoS ONE.

[26] Williamson D.L., Bigman A.C. (2018). A systematic review of medical mistrust measures. Patient Educ. Couns..

[27] Bajaj S.S., Stanford C.F. (2021). Beyond Tuskegee—Vaccine Distrust and Everyday Racism. N. Engl. J. Med..

[28] Stimpson P.J., Ortega N.A. (2023). Social media users’ perceptions about health mis- and disinformation on social media. Health Aff. Sch..

[29] Stanford C.F., Tauqeer Z., Kyle K.T. (2018). Media and Its Influence on Obesity. Curr. Obes. Rep..

[30] Park J.Y., Chung E.J., Kim N.J. (2022). Social media, misinformation, and cultivation of informational mistrust: Cultivating COVID-19 mistrust. Journalism.

[31] Nah S., Williamson D.L., Kahlor A.L., Atkinson L., Upshaw J.S., Ntang-Beb J.-L. (2023). The Roles of Social Media Use and Medical Mistrust in Black Americans’ COVID-19 Vaccine Hesitancy: The RISP Model Perspective. Health Commun..

[32] Parmar R.M., Can A.S. (2024). Dietary Approaches to Obesity Treatment.

[33] Chae K., German J., Kendrick K., Tackett S., O’Rourke P., Gudzune K.A., Laudenslager M. (2024). An obesity medicine curriculum increases the obesity care self-efficacy of internal medicine residents in the primary care setting. Clin. Obes..

[34] Conaty E.A., Denham W., Haggerty S.P., Linn J.G., Joehl R.J., Ujiki M.B. (2020). Primary Care Physicians’ Perceptions of Bariatric Surgery and Major Barriers to Referral. Obes. Surg..

[35] Bosch-Sierra N., Grau-Del Valle C., Salom C., Zaragoza-Villena B., Perea-Galera L., Falcon-Tapiador R., Rovira-Llopis S., Morillas C., Monleon D., Banuls C. (2024). Effect of a Very Low-Calorie Diet on Oxidative Stress, Inflammatory and Metabolomic Profile in Metabolically Healthy and Unhealthy Obese Subjects. Antioxidants.

[36] Ravussin E., Redman M.L. (2020). Metabolic adaptation: Is it really an illusion?. Am. J. Clin. Nutr..

[37] Gadde K.M., Atkins K.D. (2020). The limits and challenges of antiobesity pharmacotherapy. Expert Opin. Pharmacother..

[38] Wang J.-Y., Wang Q.-W., Yang X.-Y., Yang W., Li D.-R., Jin J.-Y., Zhang H.-C., Zhang X.-F. (2023). GLP−1 receptor agonists for the treatment of obesity: Role as a promising approach. Front. Endocrinol..

[39] Lincoff M.A., Brown-Frandsen K., Colhoun M.H., Deanfield J., Emerson S.S., Esbjerg S., Hardt-Lindberg S., Hovingh K.G., Kahn E.S., Kushner F.R. (2023). Semaglutide and Cardiovascular Outcomes in Obesity without Diabetes. N. Engl. J. Med..

[40] Vilar-Gomez E., Martinez-Perez Y., Calzadilla-Bertot L., Torres-Gonzalez A., Gra-Oramas B., Gonzalez-Fabian L., Friedman L.S., Diago M., Romero-Gomez M. (2015). Weight Loss Through Lifestyle Modification Significantly Reduces Features of Nonalcoholic Steatohepatitis. Gastroenterology.

[41] Prasad R., Jha K.R., Keerti A. (2022). Chronic Kidney Disease: Its Relationship with Obesity. Cureus.

[42] FDA (2024). FDA Approves First Treatment to Reduce Risk of Serious Heart Problems Specifically in Adults with Obesity or Overweight. https://www.fda.gov/news-events/press-announcements/fda-approves-first-treatment-reduce-risk-serious-heart-problems-specifically-adults-obesity-or#:~:text=Today%2C%20the%20U.S.%20Food%20and,and%20either%20obesity%20or%20overweight.

[43] Lim Z.Y., Wong J., Hussain M.S., Estee M.M., Zolio L., Page J.M., Harrison L.C., Wluka E.A., Wang Y., Cicuttini M.F. (2022). Recommendations for weight management in osteoarthritis: A systematic review of clinical practice guidelines. Osteoarthr. Cartil. Open.

[44] Fornes A., Huff J., Pritchard I.R., Godfrey M. (2022). Once-Weekly Semaglutide for Weight Management: A Clinical Review. J. Pharm. Technol..

[45] Mehta A., Marso P.S., Neeland J.I. (2017). Liraglutide for weight management: A critical review of the evidence. Obes. Sci. Pract..

[46] Balasanthiran A., Munro N., Watters K., Poots A., Morganstein D., Feher M. (2012). Liraglutide withdrawal rates: ‘real world’ practice. Pract. Diabetes.

[47] Jastreboff M.A., Aronne J.L., Ahmad N.N., Wharton S., Connery L., Alves B., Kiyosue A., Zhang S., Liu B., Bunck C.M. (2022). Tirzepatide Once Weekly for the Treatment of Obesity. N. Engl. J. Med..

[48] Kim N.T. (2020). Barriers to Obesity Management: Patient and Physician Factors. J. Obes. Metab. Syndr..

[49] Wilson R.E., Kyle K.T., Nadglowski F.J., Stanford C.F. (2017). Obesity coverage gap: Consumers perceive low coverage for obesity treatments even when workplace wellness programs target BMI. Obesity.

[50] Constantino A. Here’s How Much People Are Willing to Spend on Weight Loss Drugs, According to a New Survey. https://www.cnbc.com/2024/03/23/weight-loss-drug-cost-how-much-people-are-willing-to-spend.html#:~:text=A%20monthly%20package%20of%20a,patient%20has%20commercial%20insurance%20coverage.

[51] Kendrick N.K., Marcondes O.F., Stanford C.F., Mukamal J.K. (2022). Medicaid expansion and health care access for individuals with obesity in the United States. Obesity.

[52] Salzbrenner S.G., Lydiatt M., Helding B., Scheier L.M., Greene H., Hill P.W., McAdam-Marx C. (2023). Influence of prior authorization requirements on provider clinical decision-making. Am. J. Manag. Care.

[53] Agarwal A.A., Narayan A., Stanford C.F. (2024). Body Composition in Anti-Obesity Medication Trials—Beyond Scales. JAMA Intern. Med..

[54] OMA (2024). Leading Obesity Expert Organizations Release Statement to Patients on Compounded GLP-1 Alternatives. https://obesitymedicine.org/blog/leading-obesity-expert-organizations-release-statement-to-patients-on-glp-1-compounded-alternatives/.

[55] Eisenberg D., Shikora A.S., Aarts E., Aminian A., Angrisani L., Cohen V.R., Luca D.M., Faria L.S., Goodpaster P.S.K., Haddad A. (2022). 2022 American Society for Metabolic and Bariatric Surgery (ASMBS) and International Federation for the Surgery of Obesity and Metabolic Disorders (IFSO): Indications for Metabolic and Bariatric Surgery. Surg. Obes. Relat. Dis..

[56] Keyte R., Mantzios M., Hussain M., Tahrani A.A., Abbott S., Strachan R., Singhal R., Egan H. (2024). ‘Surgery is my only hope’: A qualitative study exploring perceptions of living with obesity and the prospect of having bariatric surgery. Clin. Obes..

[57] Stanford C.F., Kyle K.T., Claridy D.M., Nadglowski F.J., Apovian M.C. (2015). The influence of an individual’s weight perception on the acceptance of bariatric surgery. Obesity.

[58] Malhotra S., Czepiel S.K., Akam Y.E., Shaw Y.A., Sivasubramanian R., Seetharaman S., Stanford C.F. (2021). Bariatric surgery in the treatment of adolescent obesity: Current perspectives in the United States. Expert Rev. Endocrinol. Metab..

[59] Reyes C.J.K., Misra M., Lee H., Stanford C.F. (2018). Weight Loss Surgery Utilization in Patients Aged 14–25 with Severe Obesity among Several Healthcare Institutions in the United States. Front. Pediatr..

[60] Perez P.N., Stanford C.F., Chang C.D. (2020). Comment on: Socioecological factors associated with metabolic and bariatric surgery utilization: A qualitative study in an ethnically diverse sample. Surg. Obes. Relat. Dis..

[61] Stanford C.F., Mushannen T., Cortez P., Reyes C.J.K., Lee H., Gee W.D., Pratt S.J., Boepple A.P., Bredella A.M., Misra M. (2020). Comparison of Short and Long-Term Outcomes of Metabolic and Bariatric Surgery in Adolescents and Adults. Front. Endocrinol..

[62] Noria F.S., Shelby D.R., Atkins D.K., Nguyen T.N., Gadde M.K. (2023). Weight Regain after Bariatric Surgery: Scope of the Problem, Causes, Prevention, and Treatment. Curr. Diabetes Rep..

[63] Akpinar E.O., Liem R.S.L., Nienhuijs S.W., Greve J.W.M., Marang-van de Mheen P.J., Dutch Audit for Treatment of Obesity Research Group (2023). Weight recurrence after Sleeve Gastrectomy versus Roux-en-Y gastric bypass: A propensity score matched nationwide analysis. Surg. Endosc..

[64] Stanford C.F., Alfaris N., Gomez G., Ricks T.E., Shukla P.A., Corey E.K., Pratt S.J., Pomp A., Rubino F., Aronne J.L. (2017). The utility of weight loss medications after bariatric surgery for weight regain or inadequate weight loss: A multi-center study. Surg. Obes. Relat. Dis..

[65] Stanford C.F., Toth T.A., Shukla P.A., Pratt S.J., Cena H., Biino G., Aronne J.L. (2018). Weight Loss Medications in Older Adults after Bariatric Surgery for Weight Regain or Inadequate Weight Loss: A Multicenter Study. Bariatr. Surg. Pract. Patient Care.

[66] Stanford C.F. (2019). Controversial issues: A practical guide to the use of weight loss medications after bariatric surgery for weight regain or inadequate weight loss. Surg. Obes. Relat. Dis..

[67] ASMBS Estimate of Bariatric Surgery Numbers, 2011–2022. https://asmbs.org/resources/estimate-of-bariatric-surgery-numbers/.

[68] Toth A., Gomez G., Shukla A., Pratt J., Cena H., Biino G., Aronne L., Stanford F. (2018). Weight Loss Medications in Young Adults after Bariatric Surgery for Weight Regain or Inadequate Weight Loss: A Multi-Center Study. Children.

[69] Gazda L.C., Clark D.J., Lingvay I., Almandoz P.J. (2021). Pharmacotherapies for Post-Bariatric Weight Regain: Real-World Comparative Outcomes. Obesity.

[70] Anekwe V.C., Knight G.M., Seetharaman S., Dutton P.W., Chhabria M.S., Stanford C.F. (2021). Pharmacotherapeutic Options for Weight Regain after Bariatric Surgery. Curr. Treat. Options Gastroenterol..

[71] Stanford C.F., Kyle K.T. (2015). Why food policy and obesity policy are not synonymous: The need to establish clear obesity policy in the United States. Int. J. Obes..

[72] Thomas M.D., Kyle K.T., Stanford C.F. (2015). The gap between expectations and reality of exercise-induced weight loss is associated with discouragement. Prev. Med..

[73] FDA Drug Shortages. https://www.fda.gov/drugs/drug-safety-and-availability/drug-shortages.

[74] Bajaj S.S., Jain B., Kyle K.T., Gallagher C., Stanford C.F., Srivastava G. (2022). Overcoming congressional inertia on obesity requires better literacy in obesity science. Obesity.

